# Are there missed opportunities for vaccinating against hepatitis B among people who inject drugs in the UK?

**DOI:** 10.1017/S0950268819001353

**Published:** 2019-07-22

**Authors:** J. Njoroge, V.D. Hope, C. O'Halloran, C. Edmundson, R Glass, J.V. Parry, F. Ncube

**Affiliations:** 1 Public Health England, National Infection Service, Colindale, UK; 2 Public Health Institute, Liverpool John Moores University, Liverpool, UK

**Keywords:** Blood-borne virus transmission, hepatitis B, injecting drug-users (IDUs), public health, vaccines

## Abstract

Sustaining the impact of hepatitis B virus (HBV) vaccination on incidence and prevalence of HBV infection requires increasing and maintaining the uptake of vaccine among those at risk. In recent years, the level of vaccine uptake among people who inject drugs (PWID) in the UK has levelled-off. Data (2015–2016) from the national unlinked-anonymous monitoring survey of PWID, an annual survey that collects data from PWID across England, Wales and Northern Ireland, were used to examine HBV vaccine uptake. Data from participants who had injected drugs during the previous year were used to investigate sources of hepatitis B vaccine doses as well as factors associated with vaccine uptake. Among the 3175 anti-HBc-negative participants, 3138 (99%) reported their vaccination status; 23% (714) reported no vaccine uptake. Among those not vaccinated, 447 (63%) reported being sexually active and 116 (16%) reported sharing needles and syringes. Majority of those not vaccinated reported accessing services in the previous year that could have provided hepatitis B vaccine doses. These missed opportunities for vaccinating of PWID indicate a need for additional targeted interventions.

## Introduction

It is estimated that over 2 billion people worldwide have been infected with HBV, and that of these, 257 million have chronic infection [1, 2]. About 780 000 people die each year due to the consequences of hepatitis B such as cirrhosis and liver cancer [3–5]. The risk of developing chronic hepatitis B infection depends on the age at which infection is acquired. Chronic infection will develop in up to 90% of children who acquire infection before the first year of life [3]. Among adults, approximately 5–10% of those infected with HBV will develop chronic infection and 15% of those adults will develop chronic liver disease, including cirrhosis, liver failure and liver cancer [6]. Around a quarter of all liver disease cases in the UK are due to hepatitis infections [7].

When not treated, HBV is highly contagious and is transmitted through contact with infectious blood, semen or other bodily fluids through sexual contact with an infected person or through other percutaneous or permucosal exposures. People who inject drugs (PWID) can therefore be at high risk through unsafe injecting practice and often have a high prevalence of viral hepatitis. Prioritisation of PWID and also the prison population as the key groups for screening and treatment of viral hepatitis has therefore been advocated [8].

Hepatitis B is preventable with safe, effective and relatively inexpensive vaccines. A vaccine against hepatitis B virus (HBV) has been available since 1982 [4, 8, 9]. Despite this, HBV remains a major global health problem [3, 5, 10]. Although public health activities to control viral hepatitis have increased in the last three decades, interventions for prevention have not always been implemented sufficiently [2]. Since 1991, the World Health Organisation (WHO) has recommended the addition of HBV immunisation to all national immunisation programmes, but by 2004, many countries had still not implemented universal childhood immunisation [4]. Childhood HBV immunisation has since been scaled up globally and global coverage with the three doses of hepatitis B vaccine in infancy had reached 84% by 2015 [1].

Viral hepatitis continues to be a major public health threat even though new and effective treatments have been developed. Public health responses have differed between countries, and opportunities for action have not always been fully and effectively utilised. The WHO published the first global health sector strategy on viral hepatitis in 2016 which has the goal of eliminating viral hepatitis by 2030 [1]. The strategy contributes to the 2030 agenda for sustainable development [2] and covers all types of viral hepatitis but focuses on hepatitis B and hepatitis C because together these represent 95% of the viral hepatitis burden [1].

The strategy emphasises that vaccination is the key intervention for achieving the hepatitis B targets. In addition to efforts at strengthening childhood immunisation programmes, vaccine must be made available to all and uptake monitored across all population groups in all countries [1]. The UK signed up to this strategy in May 2016.

Like many other western European countries, the prevalence of HBV infection in the general population of the UK is low (<1%). Based on the overall low incidence of HBV, and with the risk of infection determined mainly by country of birth, ethnicity and adult risk behaviours such as injecting drug use, the UK has from 1988 until recently had a targeted hepatitis B vaccination programme focused on higher risk groups [7, 11, 12]. These groups include PWID and those at sexual risk (men who have sex with men and sex workers). As childhood infection accounted for an estimated 21% of all new chronic infections acquired in the UK [12], universal screening of pregnant women for hepatitis B and immunisation of babies at risk has been in place since 2000. The UK introduced a universal infant hepatitis B vaccination programme in August 2017 [13].

The UK has a well-established and extensive provision of services for people who use drugs [14]. These drug services include addiction services that offer interventions such as opiate substitution therapy (OST) and needle and syringe programmes (NSPs). These services often provide hepatitis B vaccination and blood-borne virus (BBV) testing [15]. The responsibility of commissioning of drug services has undergone changes over time notably so in England, where they are now commissioned by the local government [16].

HBV vaccine uptake among PWID has increased from 56% [17, 18] in 2004 to 72% in 2016 [19]. There has been a decline in the prevalence of anti-HBc, a marker of ever having been infected with HBV, among PWID in the UK from 29% in 2004 [17, 18] to 14% in 2016 [10]. This is probably related to the rise in vaccine uptake. The prevalence of hepatitis B surface antigen (HBsAg), a marker of current HBV infection, among anti-HBc-positive PWID has declined from 4.8% in 2011 to 3.0% in 2016 [19].

Although the uptake of vaccination among PWID is high, recent evidence suggests that this is no longer rising and may have plateaued [19]. Transmission of hepatitis B is ongoing and risky behaviours for BBV transmission, such as the sharing of needles (17%), remain common among PWID in the UK [19].

Reaching and sustaining higher levels of HBV vaccination among PWID is important in reducing transmission and HBV-related morbidity and mortality. Despite frequent contact with a range of health services that can provide hepatitis B vaccination in 2015 and 2016, a substantial proportion of PWID remained unvaccinated.

Using data from a national bio-behavioural survey for 2015 and 2016, this paper aims to examine the sources of HBV vaccine for those reporting to have ever received at least one dose of the vaccine. The factors associated with HBV vaccine uptake were also examined to explore how vaccine provision could be improved. Finally, contact with health services by those not vaccinated is explored to assess the potential missed opportunities for vaccination.

## Methods

### Sample frame, data collection and biological sample testing

PWID have been recruited into a voluntary unlinked-anonymous monitoring (UAM) survey in the UK since 1990. Methodological details of this series of annual cross-sectional surveys have been published previously [20–22]. Briefly, agencies providing services to PWID (e.g. NSPs and OST services) at sentinel locations (*N* = 67) throughout the UK, except Scotland, invite clients who have ever injected psychoactive drugs to participate in the survey. Those who consent provide a biological sample, currently a dried blood spot (DBS), and self-complete a questionnaire focused on the injection of psychoactive drugs. The UAM survey has multi-site ethics approval (NHS Health Research Authority MREC/98/2/51). In 2013, questions on the use of health services during the previous years were introduced into the survey questionnaire. Additionally, for the question asking about hepatitis B vaccine uptake, those reporting vaccine uptake were asked to indicate at which health services they received their doses of HBV vaccine.

The DBS specimens collected by the survey were tested for BBVs including the antibodies to hepatitis B core antigen (anti-HBc). For anti-HBc, an-house IgG class-specific antibody capture enzyme immunoassay (EIA) was used. Samples that were anti-HBc-positive were then tested for HBsAg.

### Data analysis

Data from 2015 and 2016 UAM survey waves were used. Participants in 2016 who reported taking part in the survey in 2015 were excluded. Only anti-HBc-negative participants who reported having injected drugs during the previous year were included in the main analyses. Among those who reported having received at least one dose of HBV vaccine, the number of doses and source of vaccine were examined.

Bivariate analyses were undertaken to establish if there was an association between the outcome variable (ever receiving a dose of hepatitis B vaccine) and a number of key explanatory variables including demographics, injecting practices and use of health services. Where possible associations were found (*P* < 0.05); these variables were further examined via multiple logistic regression using forward stepwise procedure to select variables for inclusion in the final model. All analyses were performed using SPSS 23.

For those not vaccinated, contact in the previous year with health services offering vaccination was examined to determine if there were any missed opportunities for vaccination.

## Results

### Sample characteristics

During 2015–2016, a total of 3652 participants reported injecting drugs during the preceding year (26% female, mean age 38 years). Of these, nine samples were insufficient for anti-HBc testing and 3643 samples were therefore tested, 468 (13%) of which were positive. Thus 3175 participants were anti-HBc-negative.

Of the 468 anti-HBc-positive samples, two samples were insufficient for anti-HBsAg testing, therefore 466 were tested, 14 (3%) of which were positive. Overall 14/3641 (0.38%) of the total samples tested were currently infected with hepatitis B.

Among the 3175 anti-HBc-negative participants, 3138 (99%) reported their vaccination status. In total, 2424 (76%) reported receiving at least a single dose of HBV vaccine and 714 (23%) reported that they had not been vaccinated for hepatitis B.

### Sources of hepatitis B vaccine and number of hepatitis B vaccine doses

**Fig. 1.**
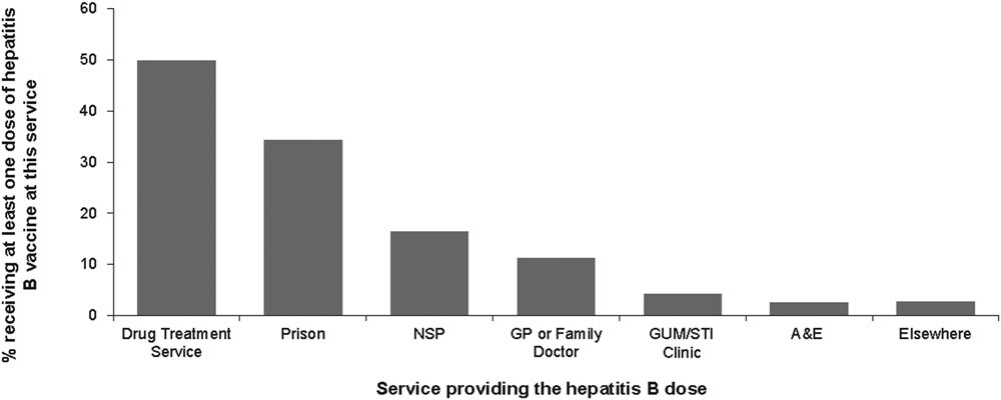
Hepatitis B vaccine uptake and the sources of hepatitis B dose.

Of the 2424 participants reporting hepatitis B vaccine uptake, 2239 (89%) reported the source of their vaccine doses. The main sources of vaccination were drug treatment services (49.9% (1117)) and prisons (34.2% (766)). Fewer participants reported receiving vaccines from the other services such as NSP (16.4% (367)), general practice (GP) or family doctor (11.3% (252)), sexually transmitted infections/genitourinary medicine (STI/GUM) clinic (4.2% (94)), emergency or casualty departments (A&E) (2.5% (57)) and hostel or homeless services (1.9% (43)) (Fig. 1).

**Fig. 2.**
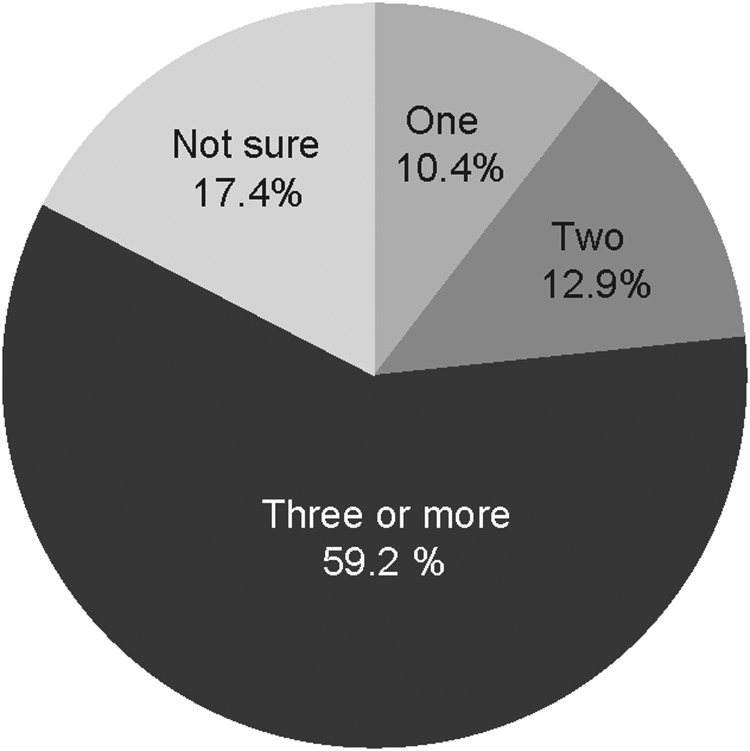
Number of hepatitis B doses received.

Of the 2239 reporting their vaccine sources, 2149 reported the number of doses (‘jabs’). Though a single dose may offer some protection, three doses are recommended to provide adequate protection. The majority (59%) of those vaccinated reported they had received three or more doses (Fig. 2).

### Factors associated with uptake of hepatitis B vaccine

In the multivariable analysis, uptake of hepatitis B vaccine varied with age with uptake being highest among those aged 40–49. Vaccination was associated with ever being imprisoned, ever been homeless, region of residence, engaging in transactional sex and being sexually active the previous year (Table 1).

**Table 1.** Factors associated with hepatitis B vaccine uptake among PWID in the UK, 2015 and 2016

Characteristic^a^	Total	Number vaccinated	(%)	*P*	Adjusted odds ratio, with 95% confidence interval		
All	3138	2424	77.2				
Demographic							
Age							
<30	566	405	71.6	<0.001	1.00		
30–39	665	499	75.0		1.034	0.785	1.361
40–50	734	599	81.6		1.446	1.088	1.921
>50	1173	921	78.5		1.215	0.938	1.574
Ever homeless							
No	741	515	69.5	<0.001	1.00		
Yes, not last year	1055	873	82.7		1.43	1.12	1.82
Yes, last year	1342	1036	77.2		1.12	0.90	1.40
Sex last year							
No	1033	766	74.2	0.004	1.00		
Yes	2105	1658	78.8		1.25	1.03	1.52
Ever been in prison							
No	1061	699	65.9	<0.001	1.00		
Yes	2077	1725	83.1		2.20	1.83	2.66
Engaged in transactional sex							
Never	2719	2074	76.3	0.003	1.00		
Yes but not in last year	209	178	85.2		1.51	1.00	2.28
Yes in last year	210	172	81.9		1.39	0.94	2.07
PHE region							
North					1.00		
Midlands and East of England					0.63	0.50	0.80
London					1.09	0.75	1.60
South					0.73	0.56	0.94
Wales and Northern Ireland					0.72	0.53	0.96
Injecting practise							
Injected heroine in the last year							
No	203	140	69.0	0.004	^b^		
Yes	2935	2284	77.8				
Health service usage last year							
Used a sexual health, GUM/STI clinic							
No	2790	2124	76.1	<0.001	1.00		
Yes	348	300	86.2		2.33	1.64	3.29
Used a GP or family doctor							
No	1007	730	72.5	<0.001	1.00		
Yes	2131	1694	79.5		1.30	1.08	1.57
Used an NSP							
No	187	102	54.5	<0.001	1.00		
Yes, not last year	310	250	80.6		2.75	1.79	4.23
Yes, last year	2641	2072	78.5		2.40	1.73	3.33
Prescribed treatment for drug use							
Never prescribed	500	299	59.8	<0.001	1.00		
Previously prescribed	509	387	76.0		1.85	1.38	2.46
Currently prescribed	2129	1738	81.6		2.50	1.99	3.13
Used A&E							
No	2120	1611	76.0	0.015	^b^		
Yes	1018	813	79.9				
Used walk-in clinic							
No	2544	1945	76.5	0.028	^b^		
Yes	594	479	80.6				

a No associations with gender, sharing injecting equipment in the last year, injected cocaine in the last year, injected speed during the preceding 12 months, injected other drugs in the preceding 12 months, visiting a family planning clinic in the preceding 12 months.

b Entered in the multivariate analyses but not included in the final model.

Regarding service usage, being vaccinated was associated with having assessed an STI/GUM clinic, seeing a GP, being prescribed treatment for drug use, with those currently prescribed treatment more likely to be vaccinated than those prescribed treatment in the past. Being vaccinated was also associated with the use of an NSP with those who had used an NSP in the past more likely to be vaccinated than those who had used the service in the previous year (Table 1).

### Contact with health services during the previous year among those not vaccinated

**Fig. 3.**
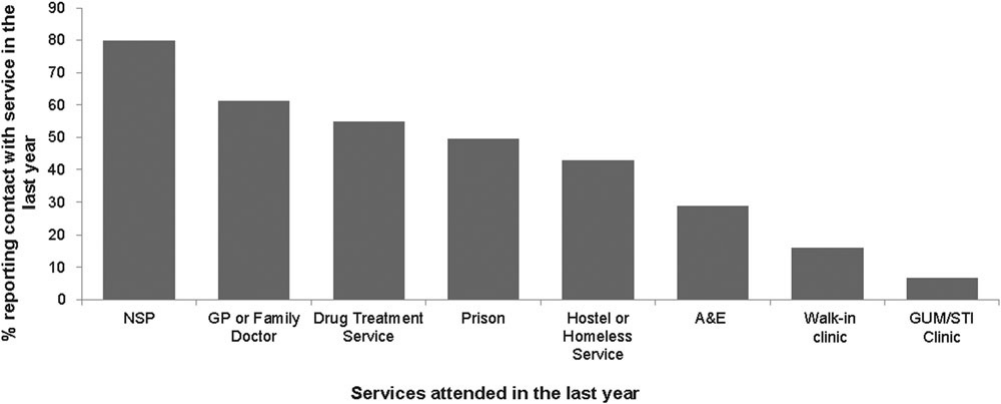
PWID in contact with service in the last year and not vaccinated for hepatitis B.

Among the 714 hepatitis B-negative participants reporting no vaccine uptake, the majority (87% (666)) had accessed at least one service in the preceding year. Most (80% (569)) had used an NSP, almost two-thirds (61% (437)) had been to a GP, over half (55% (391)) were currently in addiction treatment, almost a third (29% (205)) had attended A&E, 16% (115) had been to a walk-in clinic and 7% (48) had been to an STI/GUM clinic. In addition, about a half (49% (352)) had ever been to prison (Fig. 3).

### Sexual and injecting risks among those not vaccinated for hepatitis B

Among those unvaccinated for hepatitis B, 63% (447/714) reported being sexually active in the previous year compared with 68% (1658/2424) among those taking up the vaccine, whilst engaging in transactional sex among those unvaccinated was reported by 5.3% (38) *vs.* 7.1% (172) among those taking up the vaccine. Sharing needles and syringes in the previous year was reported by 16% of both those unvaccinated (116), and those taking up the vaccine (393). In the previous year, 91% (651) among those unvaccinated reported injecting heroin. However, injection of stimulant drugs was also common with 35% (246) injecting crack. Less commonly injected drugs were amphetamine (13% (92)), cocaine (7.6% (54)) whilst 9.4% (67) had injected other drugs.

## Discussion

### Overview

Hepatitis B vaccine uptake of 77% among PWIDs surveyed suggests that the targeted HBV vaccine approach is reaching the majority of PWID. However, in recent years, vaccination levels have plateaued and HBV transmission among PWID continues, indicating that vaccine uptake needs to increase further.

Data for 2015/16 from the National Drug Treatment Monitoring Service (NDTMS) in England show a lower level of vaccine uptake. Fifty per cent (8823/17756) among those who have ever injected drugs and potentially at risk of hepatitis B were vaccinated [19]. The difference in the reported uptake in NDTMS and the UAM survey may reflect the UAM survey data being self-reported and only for those who had injected drugs during the previous year. The UAM survey also includes those PWID who are not currently accessing addiction treatment services that report data to NDTMS. It is also possible that the data reported to NTDMS by treatment services will not fully capture the extent of vaccine uptake among their clients, for example, if vaccination has been delivered elsewhere or is not correctly recorded. Finally, NDTMS data are for England only, whereas the data from the UAM survey include Wales and Northern Ireland [19, 20].

Drug services are at the forefront of hepatitis B vaccine provision to PWID in the UK. There is evidence of a reduction in funding for the provision of drug and alcohol services for both adults and young people between 2013–14 and 2015–16 [14]. In England, local government is responsible for commissioning drug and alcohol services and receives a public health grant from central government. The drug and alcohol services element of this funding is not protected and drug and alcohol services have had to compete with other public health priorities for resources [14]. The commissioning of these services often involves regular retendering and this can disrupt service delivery. In Wales, local health boards and three NHS trusts deliver drug and alcohol services. Within the local health boards, local authorities formulate and implement strategies for drugs and alcohol. Northern Ireland has a fully integrated system for both health and social care which oversee the provision of services. As large number of organisations are involved in the commissioning of the services used by PWID, resourcing improved delivery of hepatitis B vaccine for PWID may thus be challenging.

One of the ways to increase the uptake of vaccination that has been discussed is contingency management (CM). This involves providing rewards when desired behaviour is achieved. CM can be used alone or in combination with other treatment methods and can be varied to suit the client's needs. The two common types of CM are voucher-based reinforcement and prize incentives CM [23]. In addition to increasing uptake of vaccine [24], this incentive-based approach has been found to be cost-effective [25] as well as effective in encouraging the completion of hepatitis B vaccine dose among heroin users undergoing addiction treatment [26]. Although the use of this technique has been considered and guidelines for its implementation developed [27], CM has so far not been widely implemented in the UK. The introduction of CM is potentially complex, for example, it requires the training of staff in its delivery and robust monitoring systems are needed for its effective implementation.

Our analysis also indicates that drug treatment services and prisons were the main sources of hepatitis B vaccination. In an earlier study in 2004, prison services were the most common reported sources of vaccine doses, as a result the prison vaccination programme initiated in England in 2001 [17]. Our data indicate that although the drug treatment services have overtaken prisons, prisons and NSPs remain important sources of hepatitis B vaccine provision. Prisons are important as they can reach some of those at risk of injecting before initiation. NSP can reach those recently initiated to injecting, and so have recently become at higher risk [18]. NSPs are among the first services that PWID come into contact with, with a vast majority of PWIDs in the UK reporting the first use of an NSP shortly after starting to inject [17]. It is thus of concern that in those who reported not been vaccinated, NSPs were the most commonly used service, 80% had used an NSP in the previous year.

Fewer participants reported receiving vaccination at GPs and A&E services. This may be due to PWID not disclosing their injecting drug use to health care professional when using these services due to concerns about negative responses [1, 28, 29]. Structural barriers increase vulnerability and can also prevent equitable access to services [1]. Our findings highlight an opportunity for primary and emergency care settings to engage more with the PWID who attend these services and the need to increase the offer of vaccination, and possibly the need for training to better equip health care professionals in these services to do this effectively.

Our data indicate that despite contact with services in the previous year that can offer HBV vaccination to high-risk groups, a considerable proportion of vaccine-eligible PWID missed an opportunity to be vaccinated during service contact. There therefore exists an opportunity to increase the level of vaccine delivery in all services.

### Factors associated with hepatitis B vaccine uptake

Younger participants (<30 years old) were less likely to be vaccinated and are therefore at a higher risk of acquiring hepatitis B. Younger injectors are more likely to be recent initiates to injecting and so less likely to have accessed specialist services for PWID, such as drug services, and they may therefore not have had the opportunity to be vaccinated. A higher proportion of PWID are vulnerable to hepatitis B infection during the first years of injection [12, 18]. This finding highlights the need for approaches to increase uptake among young PWIDs and to recent initiates to injecting. These could include offering vaccine at first use of NSP, or targeting the populations where the risk of initiation to injection may be elevated such as those in contact with the criminal justice system.

Vaccine uptake was associated with the region of residence, those in London being more likely to be vaccinated than those living in any of the other regions outside of London. London has a particularly high burden of hepatitis B; the incidence rate of acute hepatitis B in London in 2012 was 2.02 per 100 000 population, which is twice the national rate (England rate 1.04 per 100 000) and much higher than that seen in any of the other regions [30]. London has a higher prevalence (27%) of PWID ever infected with hepatitis B compared with the rest of England [19]. It is possible that the higher prevalence has led to larger effort resulting in a larger proportion of this risk group having been reached through targeted vaccination efforts in London.

Vaccine uptake was higher in those currently in treatment for their drug use than in those who have never been in treatment or those previously but not currently in treatment. This probably reflects the efforts to improve vaccine provision through these services. BBV testing and vaccine uptake levels are being used in the monitoring of the outcomes of drug treatment services in England, and as a result there have been recent efforts to increase BBV testing and vaccination [31]. Services have thus had BBV testing and awareness campaigns, including, for example, increased offer of testing around World Hepatitis Day. The promotion of BBV testing may also increase vaccine uptake as those testing hepatitis B-negative will be encouraged to be vaccinated.

Those in contact with NSP in the past but not currently were more likely to be vaccinated than those who had used the service during the previous year. NSPs provide a mix of services [32] and though they continue to be an important service for vaccine provision, NSPs role in providing this service might have reduced in the recent past possibly due to the recent reductions in overall funding for drug and alcohol services.

Those who had ever been to prison were more likely to have been vaccinated. The UK prison hepatitis B vaccination programme will probably have provided vaccine to many PWID and will also have reached a substantial number of people at risk of starting to inject [17]. However, our findings indicate a potential to further improve vaccine uptake in prisons. Nearly a half (49% (352)) of those who were unvaccinated reported having ever been imprisoned in the past.

An opt-out testing policy for BBVs was introduced in English prisons in 2013 [33]. As part of this policy, all prisoners found to be at risk are offered the super-accelerated hepatitis B vaccination (days 0, 7 and 21) with the first dose preferably provided on reception into the prison. Although there are concerns related to the funding of additional tests, time constraints and challenges with taking of samples, staff resources and training needs, preliminary data suggest a near doubling of BBV testing following the introduction of the opt-out testing policy [34], which may in turn lead to improved HBV vaccine uptake.

Participants reporting attending a GUM/STI clinic in the previous year were more likely to be vaccinated than those who had not used these services. This possibly reflects the routine offer of hepatitis B vaccine at these services, due to sexual contact currently being a more common transmission route for hepatitis B in the UK [12]. Sexually active PWIDs, as well as those engaged in transactional sex, were also more likely to be vaccinated, possibly reflecting higher levels of contact with sexual health services among these groups.

Although being vaccinated for hepatitis B was associated with attendance at a GP service in the previous year, there exists a potential to increase uptake at GP services as a large proportion (61% (437)) of those not vaccinated reported attending a GP service the previous year. This may be because GPs may not be correctly identifying all PWID in their population, or that PWID may not declare that they inject drugs. Additionally under the GP services contract agreement, the provision of hepatitis B vaccine to those at risk due to lifestyle or medical risk is not part of an additional list of services that GPs are required to provide [35].

### Limitations

This analysis has a number of limitations. It assumes that those who have not been vaccinated have not been offered the vaccine. It is likely that some of those not vaccinated may have been offered the vaccine but declined it. Recruiting a representative sample of PWID is difficult as there is no sampling frame for this marginalised population. To maximise representativeness, this survey used an accepted approach for surveillance surveys of PWID involving recruitment at multiple sentinel locations through services targeted at PWID [36, 37]. In the UK, there is a very extensive provision of such targeted services, and the uptake and use of these is very high, with very few of the PWID recruited through community-based studies (i.e. recruiting outside services) found not to be in contact with such services [38].

The behavioural data used here are based on self-reports. People may be unaware that they have been vaccinated or may not remember exactly what they may have been vaccinated for. The presence of antibody to the HBsAg (anti-HBs) is indicative of immunity to the HBV. Testing UAM samples for anti-HBs in the future could confirm vaccination status as reported by participants. The accuracy of behavioural data may be subject to recall bias. However, the reliability of self-reported risk behaviours among PWID has been shown in other studies [39, 40].

## Conclusion

Unvaccinated PWID remain at risk of acquiring hepatitis B. Improved and more efficient approaches to the delivery of hepatitis B vaccine to this group need to be developed and evaluated. Additional resources will probably be needed to improve uptake, but currently, obtaining these is likely to be challenging. In addition, improving performance monitoring through the development of better information systems that ensure efficient, accurate and timely recording of data within and between services, as well as developing staff skills, will be needed. As part of the overall goal to eliminate viral hepatitis by 2030, eliminating hepatitis B among PWID through vaccination coverage may be achievable, but is likely to require changes in the delivery of services for this group.
